# Thermal Infrared Face Recognition

**DOI:** 10.7759/cureus.13736

**Published:** 2021-03-06

**Authors:** Vincent A Weidlich

**Affiliations:** 1 Data Science and Artificial Intelligence, Goldsmiths, University of London, London, GBR

**Keywords:** face recognition, neural networks, artificial intelligence, deep learning, vascular map, thermal infrared imagery, long wave infrared, blood vessel pattern, facial vascular pattern, machine learning

## Abstract

The technology for deep learning in the field of thermal infrared face recognition has recently become more available for use in research, therefore allowing for the many groups working on this subject to achieve many novel findings. Thermal infrared face recognition helps recognize faces that are not able to be recognized in visible light and can additionally recognize facial blood vessel structure. Previous research regarding temperature variations, mathematical formulas, wave types, and methods in thermal infrared face recognition is reviewed.

## Introduction and background

Thermal images show great usability for facial recognition since their sensitivity to illumination changes is low. Thermal infrared (TIR) imagery of faces is nearly invariant to changes in ambient illumination [1]. Infrared image facial recognition and the required equipment has not gathered so much interest in the past, due to their cost being higher than visible video equipment, their lower image resolution, lack of datasets and high image noise. However, the advancement of infrared (IR) technology has decreased these problems. The cost of thermal cameras at the retail level has decreased considerably, making them more widely available to consumers.

**Figure 1:**
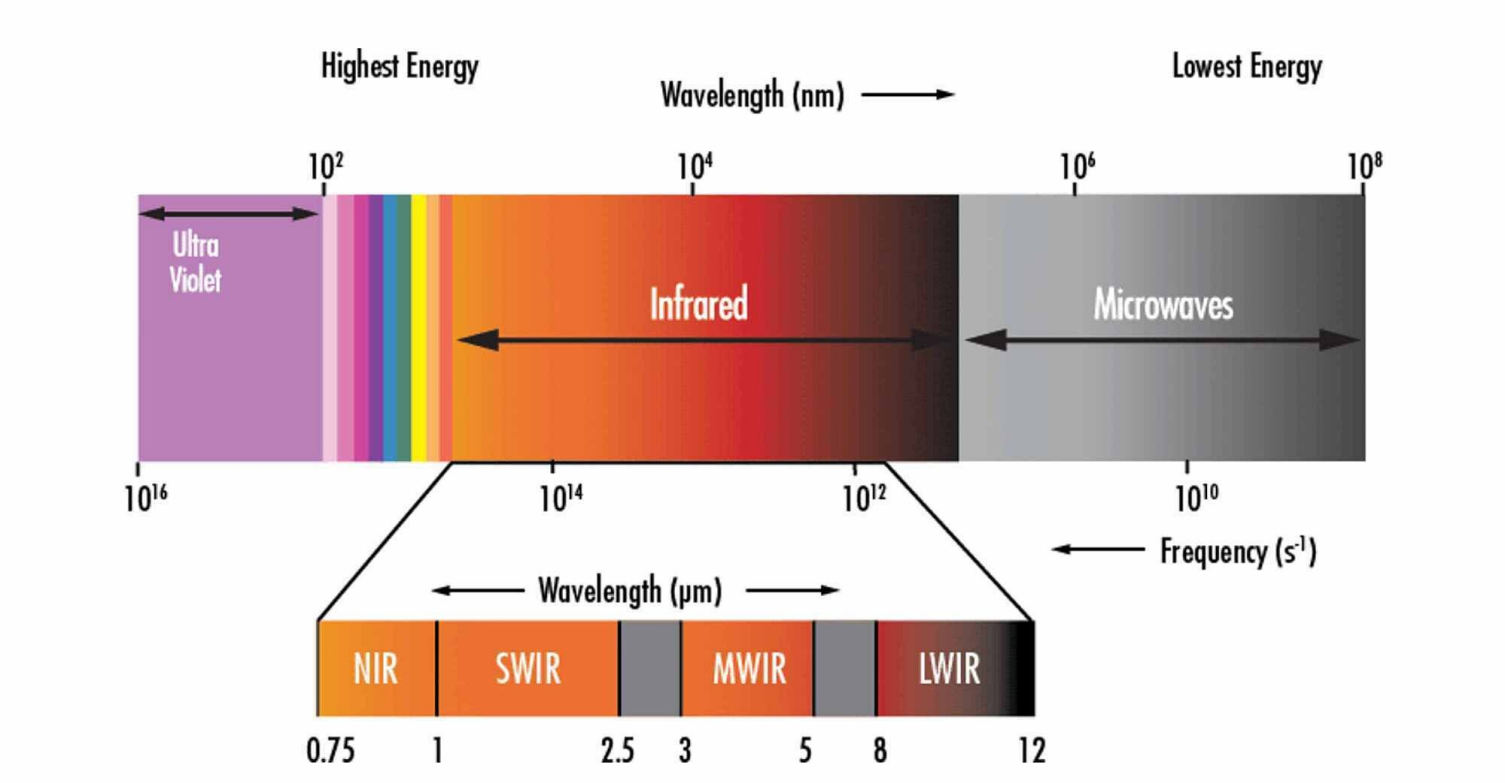
Infrared spectrum Source: [2]

The infrared (IR) spectrum can be split into the following sections as shown in Figure 1.

The human body gives off heat, which makes it possible to have a usable contrast between the body and its environment. The range of body temperature varies from 96-100 degrees Fahrenheit (F) while skin temperature in ambient room temperature varies from 79-83 F. The periorbital regions are some of the hottest areas in the human face, which allows them to be used as a feature for face tracking. TIR face recognition does, however, have difficulties in determining the eyes’ position.

## Review

Guzman et al. proposed a TIR framework for face recognition that extracts unique features and finds similarity in thermal images [1]. The framework’s protocol consisted of taking pictures of the person at four different times to allow for vascular changes over time that could affect their matching. Pavlidis et al. outlined a novel approach to the problem of face recognition in TIR [3]. The main ideas of their approach consisted of a Bayesian face detector method, followed by a physiological feature extractor. The face detector mainly uses the bimodal temperature distribution of human skin and typical indoor backgrounds. Mallat et al. discussed a novel solution based on cascaded refinement networks, which was able to generate high-quality color visible images, trained on a limited size database [4]. Their network is based on the use of contextual loss functions, enabling it to be inherently scale and rotation invariant. Gyaourova found that IR and visible imagery fusion in the wavelet domain demonstrated better recognition performance overall [5].

**Figure 2:**
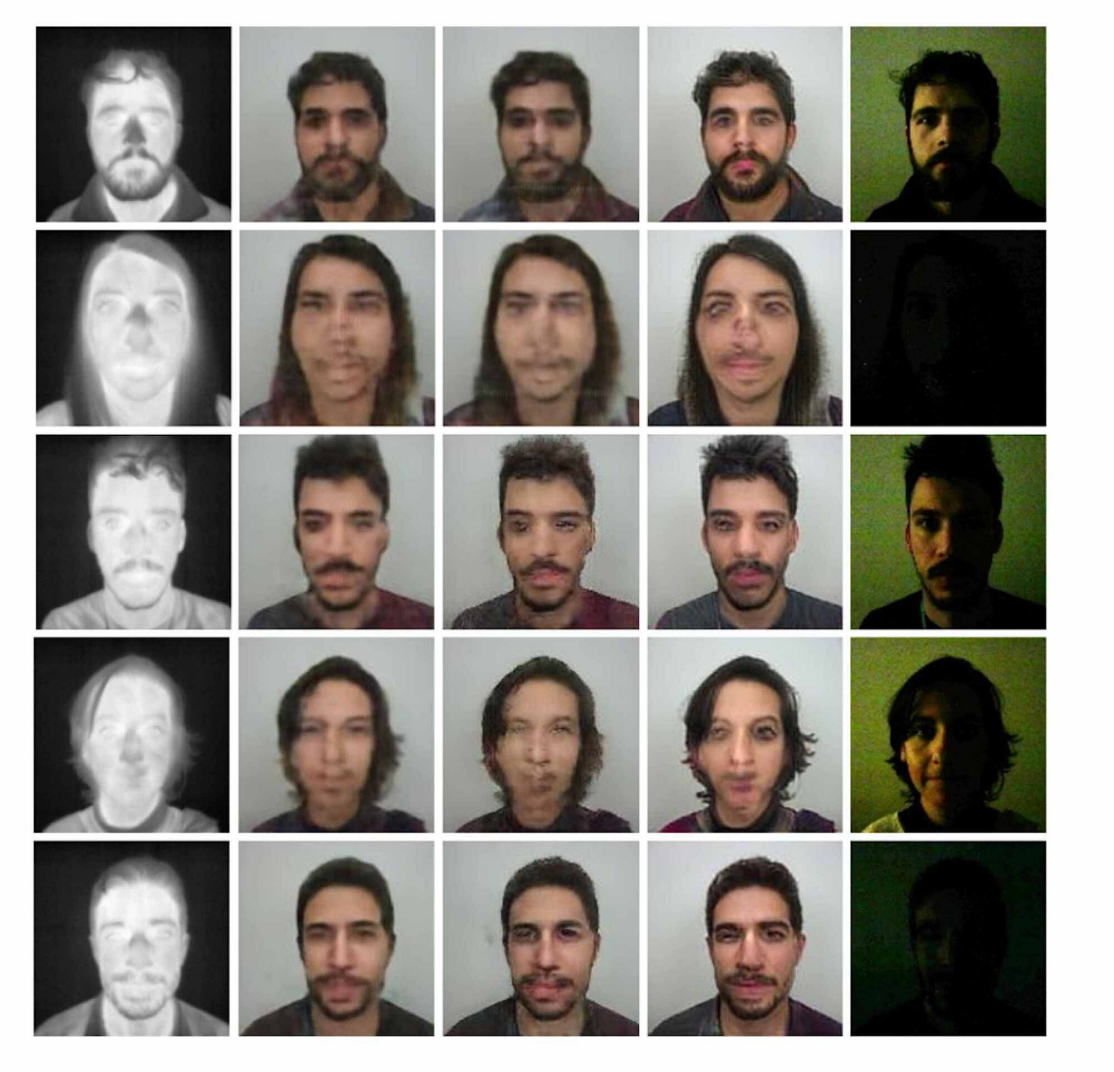
Samples of generated images acquired in total darkness Source: [4]

Figure 2 shows samples that were taken with thermal sensors in total darkness. Poor or absent illumination (shown in the right column) posed no impact on the images generated. Synthesizing images with informative facial attributes that are not in the visible spectrum was achieved [4].

**Figure 3:**
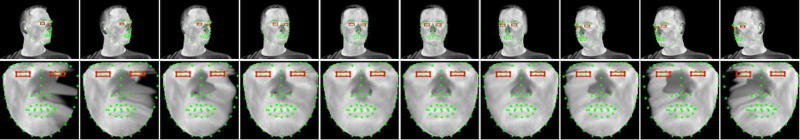
Face frontalization Note how the regions of interest (ROIs) move with the face in the original video feed but remain in a fixed position in the frontalized view. Severe out-of-plane rotation may distort the frontalized image, however, as the image shows, the amount of acceptable rotation still covers most usual head poses. Source: [6]

A common triangulation of both reference shape and detected facial landmarks makes it possible to compute a piecewise affine transformation to each triangle and apply a set of transformations to transform a face from an arbitrary position in the image into a well-defined coordinate system. This makes it possible to use fixed regions of interest (ROIs) for image analysis, even for moving faces, so additionally increasing the number of algorithms that can be applied to images with unconstrained movement. See Figure 3.

Carrapico et al. created results that showed face recognition, which could reach accuracy levels of 91% with localized binary pattern (LBP) [7]. Gabor and LBP are two well-known methods in face image analysis. The Color and Edge Directivity Descriptor (CEDD) and the Fuzzy Color and Texture Histogram (FCTH) are alternative methods that have shown good results in medical imaging. Two-dimensional Gabor filters are Gaussians weighted by an exponentially decaying sinusoid that can be applied at a given orientation and scale [8]. These filters are particularly interesting because of their behavior, resembling cells in the primary visual cortex. Selinger and Socolinsky focused their attention on longwave infrared (LWIR) imagery, in the spectral range of 8µ-12µ [9]. They studied a variety of methods in compensating for variation in illumination in order to boost recognition performance, including histogram equalization, Laplacian transforms, Gabor transforms, logarithmic transforms and 3-D shape-based methods. It is well-known that under the assumption of Lambertian reflection, the set of images of a given face acquired under all possible illumination conditions is a subspace of the vector space of images of fixed dimensions [10]. However, the set of LWIR images of a face under all possible imaging conditions is contained in a bounded set. Selinger and Socolinsky used a newly developed sensor capable of capturing simultaneous coregistered video sequences with a visible light charge-coupled device (CCD) array and LWIR microbolometer [9]. Radiometric calibration was found to provide non-uniformity correction. Socolinsky et al. saw that statistically significant evidence was presented, indicating that appearance-based face recognition algorithms applied to TIR, particularly LWIR imaging, have consistently better performance than when applied to visible imagery [11]. They found that the emissivity of the imaged object provides the further advantage of data where environmental factors contribute to a much lesser degree to within-class variability.

**Figure 4:**
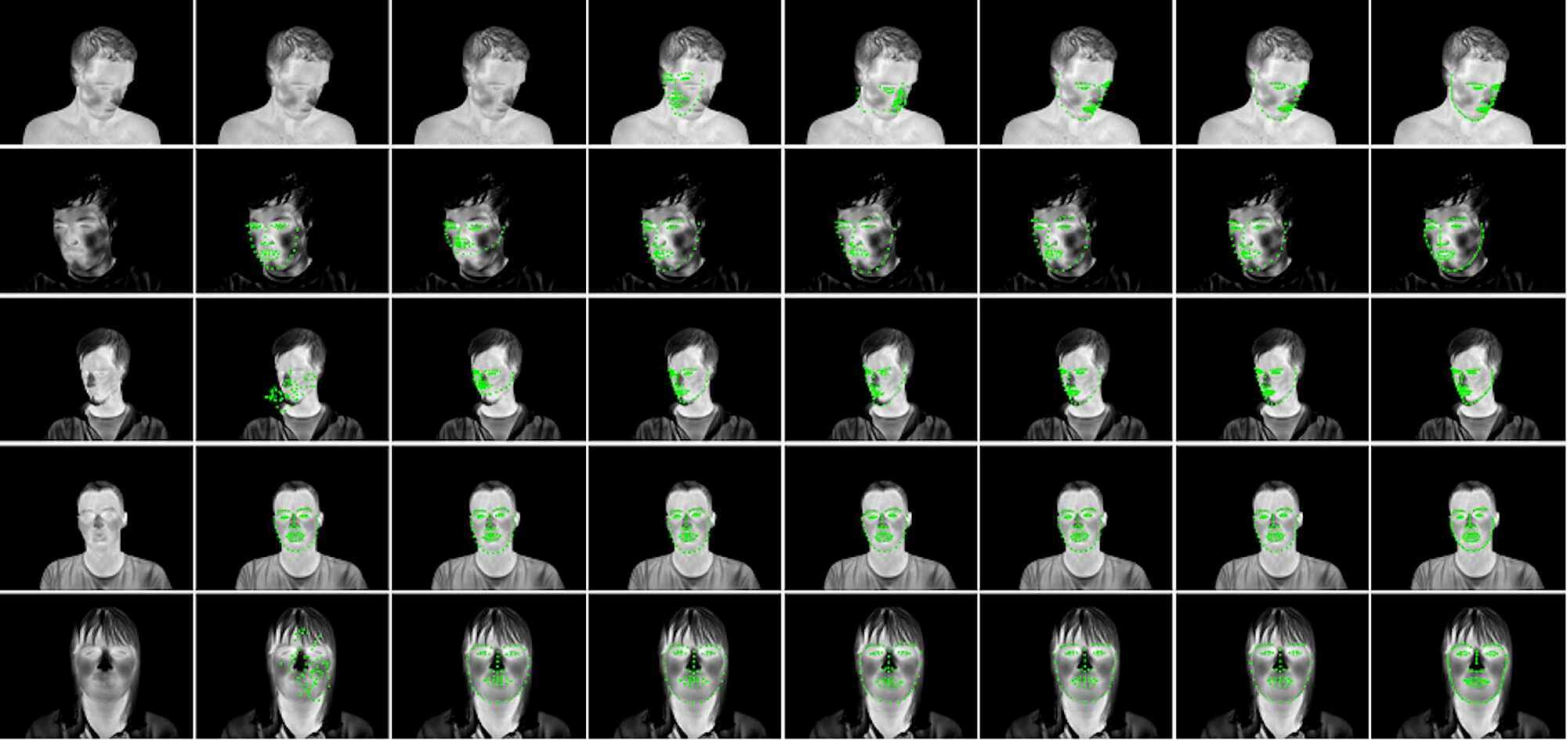
Qualitative overview of fitting performance From left to right: Original image, fast AAM version, dynamic ShapeNet, fixed ShapeNet, high-quality AAM, boundary DAN, shape-DAN, and manual ground truth. Best viewed electronically Source: [6] AAM: Active Appearance Model; DAN: Deep Alignment Network

The algorithms were parametrized using different methods to analyze the impact of algorithm modifications on tracking performance. A high-quality Active Appearance Model (AAM) parametrized for maximal precision and a high-speed AAM using a diagonal of 70 pixels were used. A Deep Alignment Network (DAN) was trained by following the results. With the trained algorithm, two different frame update strategies were implemented, which were an instance that is updated with the bounding box of the detected face (bounds-DAN) and a version that uses the detected landmark points directly for the shape update (shape-DAN). A ShapeNet was also used. The network with the database was trained, and two update strategies were evaluated, which were an instance that updates both bounding box size and position with each frame (dynamic ShapeNet) and a version that keeps a constant bounding box size and updates the face position only (fixed ShapeNet). See Figure 4.

Socolinsky et al. also found that several approaches extract thermal contours and match the shapes for identification [11]. These techniques use shape matching and the Eigenface method, which shows better results with thermal images than with visible spectrum images. They performed a comparison of recognition performance between visible and LWIR imagery based on two standard appearance-based algorithms: Eigenfaces, which are sets of eigenvectors, derived from the covariance matrix of the probability distribution over the high-dimensional vector space of face images, and Architecture for the Recognition of threats to mobile assets using Networks of multiple Affordable sensors (ARENA). Constructive Auto-associative Neural Network (CANet), which is a novel neural network inspired by neural biology, has been proposed. It is a constructive auto-associative neural network that outputs an approximation of input images using a dynamic architecture. This neural network uses receptive fields for implicit feature extraction, lateral inhibition, and auto-associative memory for image reconstruction.

Haar-based features, Constructive Auto-associative Neural Network (CANet)) and Texture Histogram offer the best results for some facial expressions [12]. Facial expression recognition using IR images has also been explored, and Trujillo proposed a facial expression recognition feature extraction model for images [13]. Principal Component Analysis (PCA) techniques, also known as Karhunen-Loeve methods, choose a linear projection that reduces the dimensionality while maximizing the scatter of all projected samples [7]. Local Feature Analysis constructs a family of feature detectors based on PCA decomposition, which is locally correlated. Haar-like transforms reduce time requirements in real-time object detection [14]. The use of Analytic Wavelet Transform (AWT) is more beneficial over Mallat wavelet transform, as AWT is translation invariant in nature [13].

**Figure 5:**
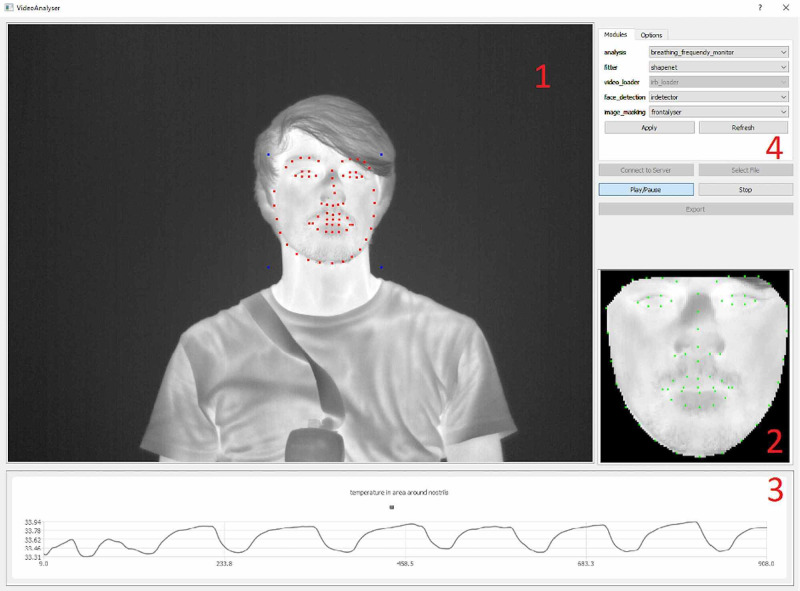
The user interface of the tracking system 1: Live video view with overlaid landmarks from the automated ShapeNet landmark detection (red) and their respective bounding box corners (blue). 2: Frontalized view of the face for improved face analysis. 3: Output of the current analysis module, in this case, a breathing rate analysis. 4: Options panel with module selection Source: [6]

Kopaczka et al. developed a system that uses a ZeroMQ-based client-server system [15]. Image acquisition and loading, face detection, facial landmark detection, frontalization, and analysis are used as distinct ZeroMQ nodes that receive their data from the server, then send it back after processing. The server forwards the data to a graphical user interface, which allows for the selecting of different modules, which can then display their results. Because of the inherent robustness of the system, all modules are designed to work independently of the others. This makes it so that a crashing module does not affect the entire system, and modules can be interchanged at run-time. See Figure 5.

**Figure 6:**
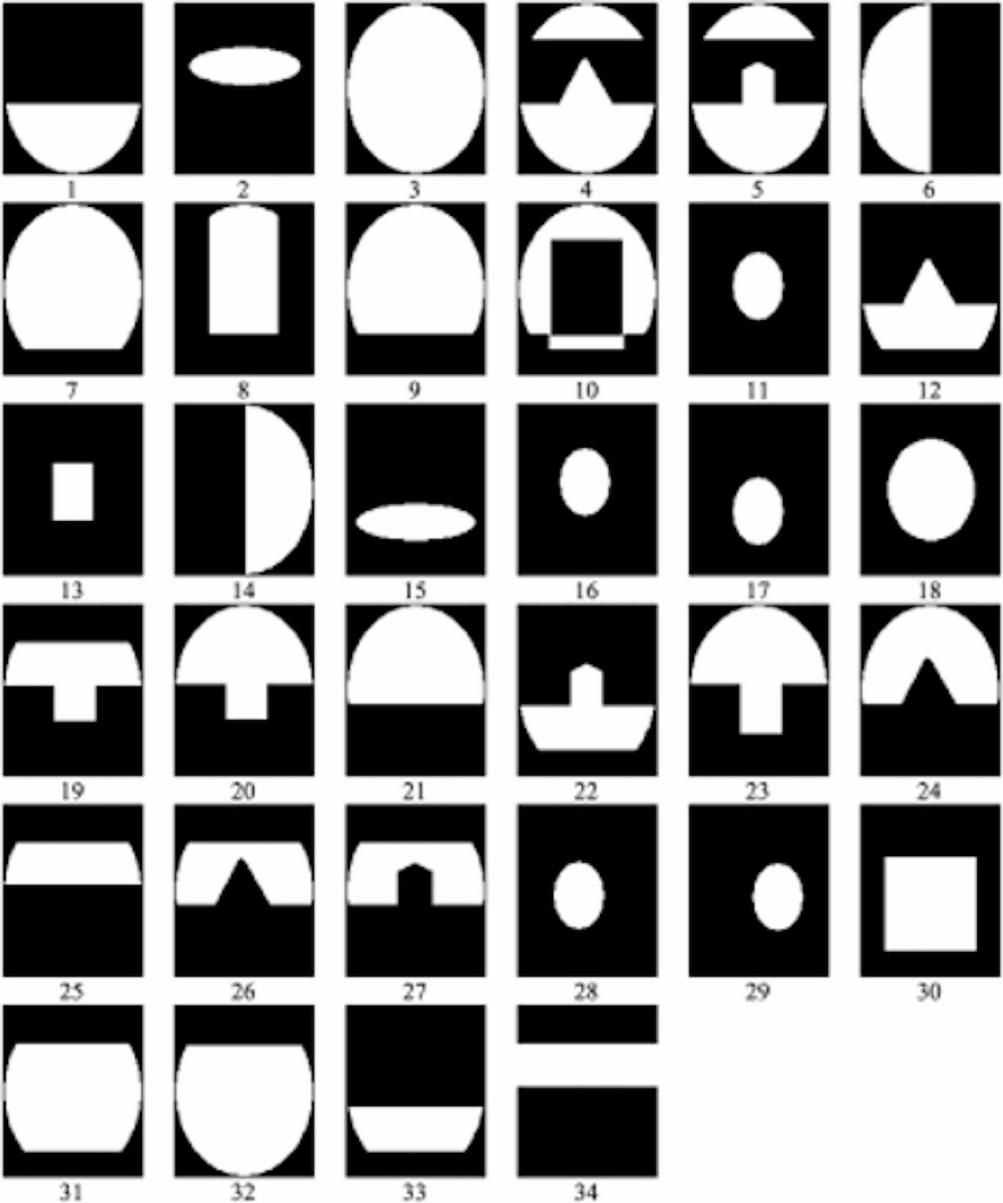
Thirty‐four local region templates Source: [16]

If looked at closely, the fused face images show spatially varying texture patterns. Instead of globally evaluating the face's surface texture pattern, it would be beneficial to consider a face image as a collection of a finite number of overlapping local regions, then classifying the face image on the basis of texture information of each individual local region. Combining all the regional classification results will make the final classification. To deal with facial occlusions on 3-D images, local analysis of facial surface was proposed, which has been used for handling facial expression variations. Thirty-four overlapping local region templates of the face were considered in Figure 6. The white area represents the considered local face region and the black area represents the excluded face region [16].

Facial skin temperature is closely related to underlying blood vessels, and the pattern of the blood vessels below the skin can be extracted by obtaining a thermal map of the human face. By performing morphological operations, like opening and top-hat segmentation to give thermal signatures, thermal feature extraction from facial images can be attained.

**Figure 7:**
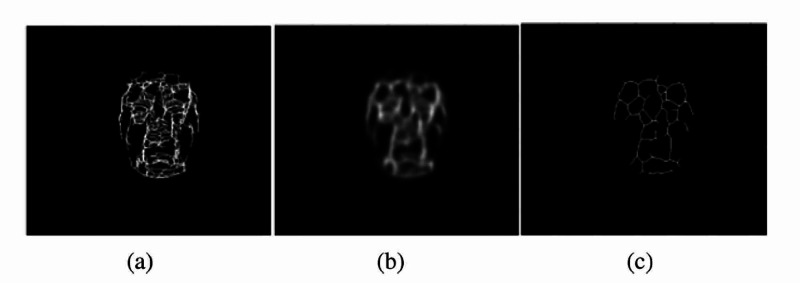
Generation of the thermal signature template (a) Resultant image of the addition of four thermal signatures. (b) Results of applying anisotropic diffusion on the summed image. (c) Thermal signature template of the subject. Source: [1]

Consistent features can be defined as features that are present in three or more thermal signatures from images taken at different times. Thermal signatures can then be extracted from each image, with the thermal signature extraction process divided into four main parts. These parts comprise face segmentation, noise removal, image morphology, and postprocessing. An individual’s thermal signatures vary from day to day because of exercise, environmental temperature, individual health, weight, imaging room temperature, and other factors. Due to these factors that can affect the thermal signature, it is recommended to establish a thermal signature template that keeps the characteristics in an individual’s thermal signature which are consistent over time. Thermal signature template generation requires the taking of extracted thermal signatures for each individual and adding them together. This creates an image, which is a composite of multiple, different signature extractions. Keeping the features that are present in all images as the dominant features that can best define the individual signature will be achieved. To fuse the predominant features, an anisotropic diffusion filter can be applied to the result of the added thermal signatures, shown in Figure 7 [1].

**Figure 8:**
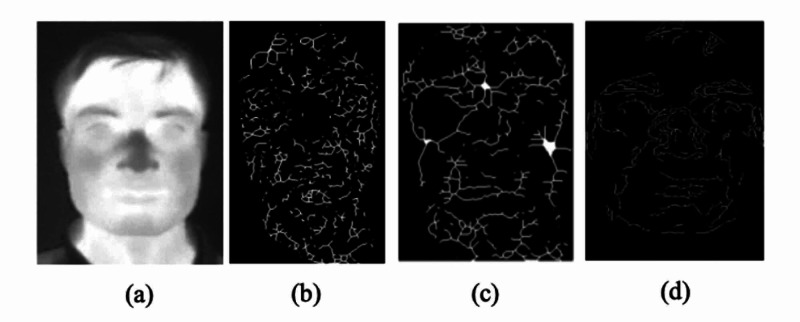
Thermal and processed images Face grayscale thermal image (a); blood perfusion obtained transforming a medial axis on bit-plane (b); morphological grey level erosion and medial axis transform (c); the result of Sobel operator (d) Source: [9]

To capture new anatomical and physiological face information, IR images can be used. This information can consist of the structure of blood vessels, facial vascular networks, and facial tissue, as well as thermal face signatures, which can be used as unique biometric features, as shown in Figure 8 [9].

**Figure 9:**
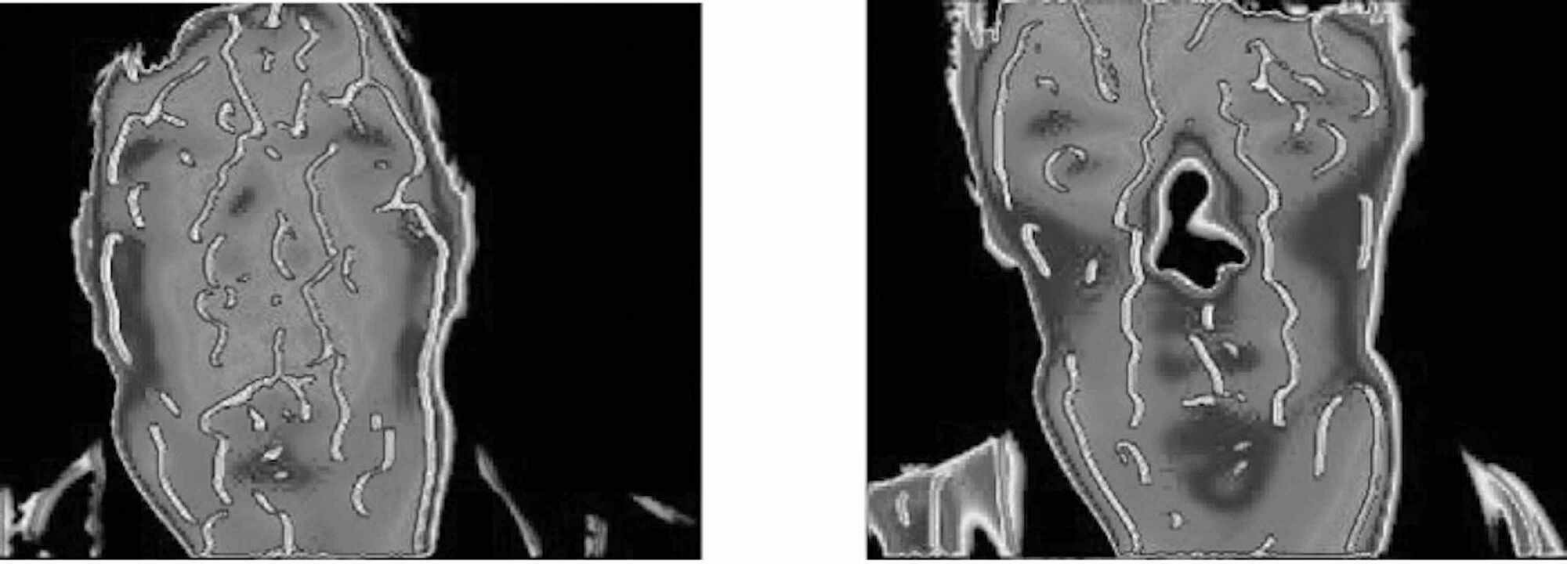
Segmented facial images annotated with the facial vascular network (white lines) Source: [3]

The process developed by Pavlidis et al. is shown, along with the facial vascular network in Figure 9 [3].

## Conclusions

Thermal imaging technology has become less expensive and more available to consumers, thus allowing more research into Thermal Infrared Face Recognition (TIR face recognition). These images can be taken with thermal sensors in total darkness. LWIR has been shown to have the best outcome in TIR face recognition. Blood vessel structure and facial vascular networks can be used as unique biometric features, thus creating a thermal map of the human face. By performing morphological operations, like opening and top-hat segmentation to give thermal signatures, thermal feature extraction from facial images can be attained.

Given the vast research that has already been performed in the field of TIR face recognition, we conclude that Long Wave Infrared can be used to create thermal images of face feature maps and facial vascular maps in order to identify individuals. This technology promises to be a viable alternative to visible light face recognition methods and technologies, thereby allowing the critical function of face recognition to be performed independently of ambient illumination conditions. Furthermore, due to the more detailed and larger amount of facial features detectable with TIR, this method is expected to differentiate more quantitatively available facial features compared to visible light face recognition. TIR face recognition is therefore expected to be more precise than visible light face recognition. Based on these principal findings, much developmental research remains to be performed to apply the stated advantages in practice and provide reliable, precise, and user-friendly TIR face recognition solutions.
